# Plasmodium falciparum Regulatory Subunit of cAMP-Dependent PKA and Anion Channel Conductance

**DOI:** 10.1371/journal.ppat.0040019

**Published:** 2008-02-01

**Authors:** Anaïs Merckx, Marie-Paule Nivez, Guillaume Bouyer, Pietro Alano, Gordon Langsley, Kirk Deitsch, Serge Thomas, Christian Doerig, Stéphane Egée

**Affiliations:** 1 INSERM U609, Wellcome Center for Molecular Parasitology, Glasgow Biomedical Research Centre, Glasgow, Scotland, United Kingdom; 2 Institut Cochin, INSERM U567, Université Paris Descartes, CNRS (UMR 8104), Paris, France; 3 Université Pierre et Marie Curie – CNRS UMR 7150, Roscoff, France; 4 Instituto Superiore de Sanita, Rome, Italy; 5 Department of Microbiology and Immunology, Weill Medical College of Cornell University, New York, New York, United States of America; University of Connecticut Health Center, United States of America

## Abstract

Malaria symptoms occur during Plasmodium falciparum development into red blood cells. During this process, the parasites make substantial modifications to the host cell in order to facilitate nutrient uptake and aid in parasite metabolism. One significant alteration that is required for parasite development is the establishment of an anion channel, as part of the establishment of New Permeation Pathways (NPPs) in the red blood cell plasma membrane, and we have shown previously that one channel can be activated in uninfected cells by exogenous protein kinase A. Here, we present evidence that in P. falciparum-infected red blood cells, a cAMP pathway modulates anion conductance of the erythrocyte membrane. In patch-clamp experiments on infected erythrocytes, addition of recombinant PfPKA-R to the pipette *in vitro*, or overexpression of PfPKA-R in transgenic parasites lead to down-regulation of anion conductance. Moreover, this overexpressing PfPKA-R strain has a growth defect that can be restored by increasing the levels of intracellular cAMP. Our data demonstrate that the anion channel is indeed regulated by a cAMP-dependent pathway in *P. falciparum-*infected red blood cells. The discovery of a parasite regulatory pathway responsible for modulating anion channel activity in the membranes of P. falciparum-infected red blood cells represents an important insight into how parasites modify host cell permeation pathways. These findings may also provide an avenue for the development of new intervention strategies targeting this important anion channel and its regulation.

## Author Summary

By replicating within red blood cells malaria parasites are largely hidden from immune recognition, but within mature erythrocytes nutrients are limiting and accumulation of potentially hazardous metabolic end products can rapidly become critical. In order to survive within red blood cells malaria parasites, therefore, alter the permeability of the erythrocyte plasma membrane either by up-regulating existing carriers, or by creating new permeation pathways. Recent electrophysiological studies of *Plasmodium*-infected erythrocytes have demonstrated that these changes reflect trans-membrane transport through ion channels in the infected erythrocyte plasma membrane. Protein phosphorylation has been documented in protozoan parasites for a number of years and is implicated in key processes of both parasites and parasitized host cells. It has been established that cAMP-dependent regulated pathways are able to activate ion channels in the red cell membrane and a better understanding of how the parasite manipulates cAMP-dependent signaling to activate anion channels could be important in developing novel strategies for future anti-malarial chemotherapies.

## Introduction

Plasmodium falciparum is the species responsible for the lethal form of malaria [1]. The disease is caused by the developmental cycle of the blood stage of the parasite [2]. Their proliferation within the red blood cells (RBCs) requires amino acids from the degradation of haemoglobin, but it also depends on the uptake of essential nutrients from blood plasma such as panthotenate or glutamate, whereas waste products such as lactate have to be extruded. These compounds are imported/exported via constitutively active endogenous transporters and new parasite-induced permeation pathways (NPPs) [3]. NPPs appear about 12–15 h after red blood cell invasion by merozoites and increase noticeably reaching a plateau 36 h post-invasion [4]. The substrate specificities of NPPs have been extensively characterized by means of tracer fluxes and iso-osmotic haemolysis experiments and show permeability for monosaccharide sugars and other small polyols, amino acids, peptides, nucleosides, various monocarboxylates, small quaternary ammonium compounds, and monovalent inorganic anions and cations including Ca^2+^ (for review [5,6]). Furthermore, they have been found to be non-saturable, to have an activation energy lower than that typical of carrier-mediated transports and to be blocked by numerous anion transport inhibitors (e.g. Furosemide and 5-nitro-2(3-phenylpropylamino) benzoic acid (NPPB)) leading to the conclusion that NPPs have some of the characteristics of anion channels [6].

It has been previously shown that after invasion, at the trophozoite stage, anion conductances are activated in the red cell membrane [7–9]. Even if the number and the origin (human or parasite encoded) of the channels responsible for this increase in membrane conductance are still under debate [10–12], today there appears to be a consensus that membrane conductance of infected cells is predominantly inwardly rectified and due to the activity of anion channels [11]. Moreover, recent data showed that this inwardly rectified membrane conductance can be accounted for by the activity of two different types of channels [13]. However, whether or not these channels are indeed correlates of NPPs already described by fluxes and semi-quantitative haemolysis experiments has not been established [11]. Nevertheless, in a recent attempt to unify the different data provided by flux experiments, semi-quantitive haemolysis experiments and results reported using the whole-cell configuration of the patch-clamp technique, it was shown that solute transport via NPPs is not consistent with a single channel model [10,11]. For example, sorbitol does not enter infected RBCs via a channel with a low open-state probability at positive membrane potential.

We have previously shown using cell-attached and inside-out configurations of the patch-clamp technique that one of the anion channels described at the single channel level could be activated in uninfected erythrocytes by stimulation of the cAMP-dependent signalling pathway, while in infected red blood cells this anion channel is activated by the parasite [8,14]. Moreover, this increase in membrane conductance following parasite infection can be inhibited by intracellular dephosphorylation [15,16].

The cAMP-dependent signalling pathway has attracted interest from a number of groups [17–19] and cAMP-dependent protein kinase catalytic subunit (PKA-C) homologues have been reported in P. yoelii [20] and P. falciparum [21,22]. The cAMP pathway plays a central role in many developmental processes in eukaryotes [23–25]. It is generally activated by the binding of a ligand to a membrane receptor, which then causes dissociation of heterotrimeric G-proteins. One of the monomeric G-proteins activates a membrane-bound adenylate cyclase, which causes an increase in intracellular cAMP concentration. In un-stimulated cells, PKA-C is bound to an inhibitory regulatory subunit (PKA-R) in a tetrameric complex composed of two PKA-R and two PKA-C molecules. Binding of cAMP to the regulatory subunits, each of which contains two cAMP-binding sites, releases the PKA-C subunits, which results in their activation. PKA-C substrates include transcription factors and other proteins involved in developmental processes [17].

PKA activity has been detected in P. falciparum asexual blood-stage parasites and is higher in mature schizonts than in younger stages of parasite development [22]. Treatment of infected cultures with H89, a chemical inhibitor of PKA, inhibits parasite proliferation [22]. It is unclear however, whether the unique target is the PKA of the parasite, that of the host red blood cell, or another enzyme altogether (H89 inhibits other protein kinases in addition to PKA) [26]. Some residues of mammalian PKA-C that are important for binding to the regulatory subunit are not conserved in the PKA-C subunits of P. yoelii and P. falciparum. Biochemical evidence for the presence of cAMP-binding proteins in parasite extracts, which may include parasite PKA regulatory subunits, has been reported [27]. Moreover, cAMP has also been implicated in regulation of the parasite's cell cycle [25].

In addition to PfPKA-C, which is the only PKA-C subunit among all the P. falciparum kinases (commonly called the P. falciparum kinome) [28], nucleotide cyclases [29] and phosphodiesterases [30] have also been described in P. falciparum. To complete the picture of the cAMP pathway in the parasite, we characterised the single PKA-R subunit of P. falciparum (PfPKA-R), and demonstrate that this protein interacts with a cAMP-inducible kinase activity in P. falciparum extracts. Finally, we show that exogenous recombinant PfPKA-R, as well as overexpression of PfPKA-R in transgenic parasites, affects electrophysiological properties of the anion membrane conductance of infected erythrocytes.

## Results

### Identification of PfPKA-R

**Figure 1:**
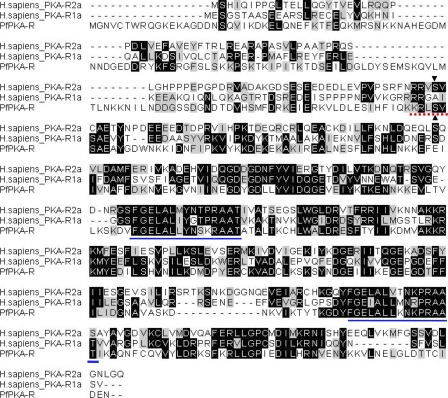
Alignment of the PfPKA-R Protein Sequence with That of Human PKA Regulatory Type Iα and Type IIβ Subunits The conserved cyclic nucleotide binding motifs are underlined in blue, and residues involved in interaction of mammalian PKA-R with the catalytic subunit (the inhibitory sequence) are underlined in red, and the phosphorylable serine is indicated by black arrows.

BLASTP analyses of predicted P. falciparum proteins in the PlasmoDB database using PKA-R subunits from various eukaryotes as queries identified a single P. falciparum polypeptide displaying significant homology to the query sequences, in agreement with our previous study [28]. The predicted 441-residue protein, which we call PfPKA-R (PlasmoDB identifier PFL1110c, or GenBank [http://www.ncbi.nlm.nih.gov/]/EBI Data Bank [http://www.ebi.ac.uk/embl/] accession number AJ441326), displays 42.7% and 47.7% similarity to human IIα and Iα PKA regulatory subunits, respectively (Figure 1), and contains a Pfam cNMP domain (PF00027) that encompasses two pairs of the PROSITE cNMP-binding motifs (PS00888 and PS00889). More specifically, PfPKA-R contains two cyclic nucleotide binding site signatures (Figure 1), called Phosphate Binding Cassettes, that specifically identify proteins belonging to the R-subunit PKA family [31]. PfPKA-R does not appear to possess a dimerisation domain and shows very weak homology to known PKA-R in the C-terminal part of the protein. Overall, P. falciparum PKA-R is most similar to the human RI subunit, but by the presence of a phosphorylatable residue (serine) in the hinge region (K_146_RLSV_150_), PfPKA-R also resembles the RII subunit found in higher eukaryotes [32] (see Figure S1). Thus, the single PKA-R subunit present in P. falciparum shares characteristics with both mammalian RI and RII subunits (Figure 1). Sequence analysis of the *pfpka-r* coding region amplified from a cDNA library confirmed the annotation gene prediction proposed in PlasmoDB (http://www.PlasmoDB.org). Northern blot analysis detected a 2.2 kb *pfpka-r* transcript (see below), with higher levels in asexual parasites compared to gametocytes. We have previously reported a similar pattern for *pfpka-c* mRNA [22].

**Figure 2:**
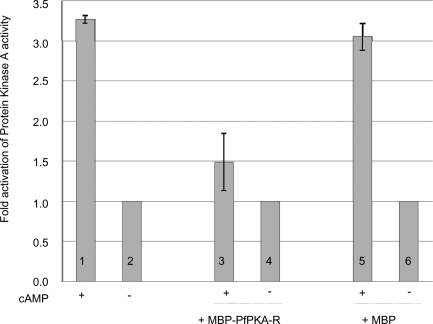
Fold of Activation of a Kemptide-Kinase Activity in Parasite Extracts Following Addition of cAMP, and in Presence of MBP-PfPKA-R and MBP (as a Control) PKA activity has been normalised to Kemptide-kinase activity in absence of cAMP and was measured as described in Methods.

To determine whether PfPKA-R is able to modulate a cAMP-dependent kinase activity, PfPKA-R was expressed as a Maltose-Binding Protein (MBP) fusion protein and incubated with parasite extracts in the presence or absence of cAMP. As a control we incubated the MBP moiety alone with parasite extracts. A kinase reaction was then performed and ^32^P incorporation into Kemptide was monitored (Figure 2). PKA activity was normalised to Kemptide-kinase activity in absence of cAMP. There is a significant difference in activity when the kinase assays are performed in presence compared to the absence of cAMP in parasite extracts (columns 1 and 2, *p* = 0.0002, *n* = 2), as we previously reported [22], and compared to parasite extracts incubated with only the MBP moiety (columns 5 and 6, *p* = 0.0032, *n* = 2) showing that MBP has no significant effect on PKA activity on its own. In contrast, addition of MBP-PfPKA-R causes a 55% decrease in the cAMP-dependent Kemptide kinase activity present in the parasite extract (columns 3 and 4, *p* = 0.1524, *n* = 2), suggesting in this case that the cAMP interacts with the endogenous PfPKA-R and with the recombinant MBP-PfPKA-R.

### PKA Regulation of Anion Transport across the Infected Erythrocyte Membrane

**Figure 3:**
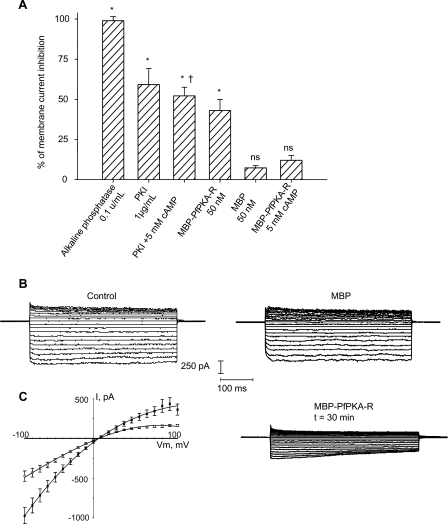
Patch Clamp Experiments on Infected Erythrocytes (A) Membrane current inhibition expressed as a percentage, in presence of 0.1 U/ml alkaline phosphatase (*n* = 6), 1 μg/ml PKI with and without 5 mM cAMP (*n* = 4), 50 nM MBP-PfPKA-R with and without 5 mM cAMP (*n* = 6), and in presence of MBP (*n* = 6). Percentage of membrane current inhibition were established when the effect of the drug reached the maximum and calculated against the values of membrane currents obtained directly after patch rupture and whole-cell achievement. All daily measurements were compared to non-treated cells. In all cases, *n* denotes the number of cells tested. ∗, *p* < 0.05 paired, two-tailed t-test relative to the control; ns, not significantly different; †, *p* > 0.05 between the conditions with PKI in presence or absence of cAMP. (B) Typical current traces obtained in the presence of MBP in the pipette (negative control). (C) Current traces in presence of 50 nM MBP-PfPKA-R (□, *n* = 6) and corresponding I-V plots with control cells (MBP alone, , *n* = 6) (mean ± standard error of the mean).

The whole-cell configuration of the patch-clamp technique allows one to follow changes in membrane conductance after erythrocyte infection. Although this configuration gives activity measurements of all channels present in the red blood cell membrane, it is limited experimentally, as one cannot change the cytosolic medium during the time course of recordings. For this reason, in this study non-diffusible compounds were added to the pipette solution and the effects were compared to control cells tested the same day in parallel. An inwardly rectified whole cell current is inducible in uninfected erythrocytes by addition of bovine PKA and ATP, and the resulting membrane currents are similar in shape to those measured in P. falciparum-infected red blood cells [8]. The addition of alkaline phosphatase (0.1 u/ml) results in a complete inhibition of current, suggesting regulation by phosphorylation (Figure 3). Thus, in order to test the implication of a cAMP-dependent kinase, we added the natural inhibitor of mammalian PKA, PKI (1 μg/ml), into the pipette. PKI is a small heat-stable protein that is highly specific (Ki = 2.3 nM) [33] interacting with the mammalian catalytic subunit within the cleft (between the two lobes) and with the larger lobe of the enzyme [34].

Maximum inhibition of current is reached after 10–15 minutes and corresponds to 59.2 ± 10.0% of membrane currents inhibition (*n* = 4). As expected, according to the mode of inhibition of PKA by PKI in mammals, this inhibition could not be reversed by competition with exogenous 5 mM cAMP, suggesting a direct interaction between the PKI and the PKA-C target (Figure 3A).

We next investigated whether or not addition of recombinant PfPKA-R into the pipette could repress the P. falciparum-induced membrane currents. Addition of MBP-PfPKA-R down-regulates whole-cell membrane conductance in a time-dependent manner (Figure 3C). Maximum inhibition of membrane current is 43.0 ± 7.0% (*n* = 4), whereas MBP alone did not change the shape of currents, nor membrane conductance during at least 30 minutes (Figure 3B). Moreover, the down-regulation appears to be regulated by cAMP, as addition of 5 mM cAMP resulted in <10% inhibition (versus >40% in the absence of cAMP, see Figure 3A). These results demonstrate that addition of exogenous PfPKA-R interferes with the parasite-dependent activation of infected red blood cell membrane conductance.

**Figure 4:**
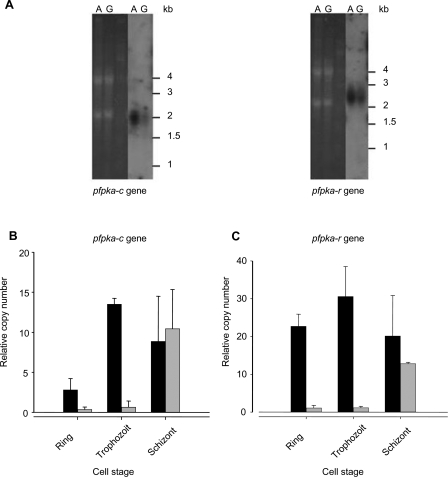
*pfpka-c* and *pfpka-r* Transcription Profiles in Wild-Type and PfPKA-R Overexpressing Parasites (A) Ethidium bromide staining and northern blot analysis on 3D7 asexual asynchronous parasites (lanes A) and Percoll purified 3D7 gametocytes (stages III and IV, 95% pure) (lanes G) for *pfpka-c* (left panel) and *pfpka-r* (right panel) transcripts. (B) Analysis of the level of transcription profile of *pfpka-c* and (C) *pfpka-r* genes in transformed pHL-*dhfr-luciferase* (grey bars) and pHL-*dhfr-pfpka-r* (black bars) cell lines. Data were calculated from triplicated data from two different RNA extractions. All values are presented as relative copy number to the housekeeping gene *seryl-tRNA synthetase* (PF07_0073).

**Figure 5:**
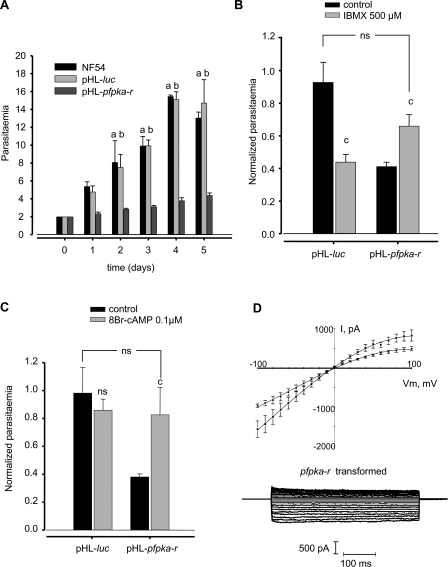
Growth and Membrane Anion Conductance of PfPKA-R Overexpressing Parasites (A) Growth of the NF54 wild type (black bars), *luciferase* (grey bars) transformed parasites, and *pfpka-r* (dark grey bars) transformed parasites. The parasitemia (percentage of red cells infected) was determined by counting infected cells (1,000) from three different experiments. Initial parasitemia was 2%, and the maximal parasitemia was around 15%. (a) and (b) denote respectively, statistical differences (*p* < 0.05) between parasitaemia of NF54 (a) strain and pHL-*dhfr-luciferase* transformed parasites (b) with PfPKA-R overexpressing parasites (*n* = 3 for each condition). (B) Effect of the phosphodiesterase inhibitor IBMX (500 μM) on the growth of *pfpka-r* transformed parasites. Data are expressed as the mean normalized parasitemia compared to the control NF54 parasites without treatments (± standard error of the mean; *n* = 3). Experiments were realized by propagating synchronized cultures in normal medium (black bar) or in medium supplemented with 500 μM IBMX (grey bars) during 72 h. In these experiments the initial parasitemia was 2%. c, *p* < 0.05 paired, two-tailed t-test relative to the parasites without drug; ns, not significantly different between pHL-*dhfr-luciferase* parasites and IBMX treated *pfpka-r* parasites. (C) Effect of the cell permeable analogue of cAMP (8Br-cAMP, 0.1 μM) on the growth of *pfpka-r* transformed parasites. Data are expressed as the mean normalized parasitemia compared to the control NF54 parasites without treatments (±standard error of the mean; *n* = 3). Experiments were realized by propagating synchronized cultures in normal medium (black bar), or in medium supplemented with 0.1 μM 8Br-cAMP (grey bars) during 72h. In these experiments the initial parasitemia was 2%. c, *p* < 0.05 paired, two-tailed t-test relative to the parasites without drug; ns, not significantly different between pHL-*dhfr-luciferase* parasites and 8Br-cAMP treated *pfpka-r* parasites. (D) Current traces recorded on PfPKA-R overexpressing parasite-infected erythrocytes (▪, *n* = 6) and corresponding I-V plots and pHL-*dhfr*-*luciferase* transformed cells as control (, *n* = 6) (mean ± standard error of the mean). In all cases, *n* denotes the number of cells tested.

To investigate PfPKA-R function *in vivo*, we generated a P. falciparum line that overexpresses the protein from the pHL-*dhfr-pfpka-r* vector. Using parasites transfected with pHL-*dhfr-luciferase* as a control, we assessed the expression of the *pfpka-r* transgene. The expression occurs in asexual stages, as observed in wild type parasites (Figure 4A), but was high and constant throughout the erythrocytic cycle (Figure 4C), as expected for a transgene driven by the *hrp3* promoter [35]. In wild-type and in pHL-*dhfr-luciferase*-transfected control parasites, expression of *pfpka-c* and *pfpka-r* peaks at the schizont stage, with very low levels in earlier stages of the asexual cycle (Figure 4B and 4C and http://www.plasmodb.org/). In contrast, the PfPKA-R overexpressers produced large amounts of *pfpka-c* transcripts at the ring and trophozoite stages (Figure 4B). This might be explained by the parasite's attempt to compensate any deleterious effects stemming from overexpression of the regulatory subunit. Growth of PfPKA-R-overexpressing parasites was monitored for five days. While the growth rate of pHL-*dhfr-luciferase* transgenic parasites was similar to that of the parental wild type NF54 strain (*p* = 0.6, *n* = 3 after five days, Figure 5A), overexpression of PfPKA-R reduced parasite growth rate by 78 ± 3% after five days (*p* < 0.05, *n* = 3) (Figure 5A). In order to check if reduced growth was due to dampened cAMP signalling, we monitored the growth of NF54 (and of NF54 transformed either with pHL-*dhfr*-*pfpka-r*, or pHL-*dhfr-luciferase*) in the presence of a phosphodiesterase inhibitor (IBMX). Addition of IBMX (500 μM) to the culture medium caused a rise in cAMP levels in control infected cells and in parasites overexpressing PfPKA-R from 0.20 ± 0.03 pmol/10^8^ cells to 0.82 ± 0.05 pmol/10^8^ cells (*p* < 0.05, *n* = 2) and from 0.60 ± 0.20 pmol/10^8^ cells to 1.40 ± 0.34 pmol/10^8^ cells (*p* < 0.05, *n* = 2) respectively, as previously described [25]. This partially restored the growth rate of parasites overexpressing PfPKA-R, reaching 66% ± 7 (*n* = 3) of the normalized parasitaemia of non-transformed NF54 parasites (*p* > 0.1, *n* = 3, Figure 5B). However, IBMX stimulation of the pHL-*dhfr-luciferase* transformed parasites caused a dramatic fall of the growth to 43% ± 6 of the normalized parasitaemia of non-transformed NF54 parasites (*p* < 0.05, *n* = 3, Figure 5B). A similar phenomenon is observed in non-transformed NF54 parasites (data not shown). Moreover, addition of the permeable analogue of cAMP (8Br-cAMP, 0.1μM), enhanced the growth of the PfPKA-R overexpressing parasites reaching 82 ± 19% (*n* = 3) of the normalized parasitaemia of non-transformed NF54 parasites (*p* = 0.6, *n* = 3, Figure 5C). Stimulation by exogenous 8Br-cAMP of pHL-*dhfr-luciferase* transformed parasites caused a decrease in growth (Figure 5C) that was not significant (*p* = 0.6, *n* = 4), as previously seen with IBMX. Together, these results suggest that the level of intracellular cAMP has to be finely tuned in order to ensure normal cell cycle and differentiation.

The PfPKA-R overexpressor and control parasite populations were analysed using the whole-cell patch-clamp method. While the luciferase expressing population showed membrane currents similar to those found in non-transfected cells, the population overexpressing PfPKA-R presented a clear reduction of membrane currents (40.4 ± 6.6%, *n* = 6) (Figure 5D and data not shown). The inhibition was similar in amplitude to that observed when the MBP-PfPKA-R recombinant protein was added to the patch pipette (see Figure 3A and 3C). Interestingly, 24 h stimulation of the overexpressing PfPKA-R cells, in the presence of IBMX, restored the membrane conductances (Figure S2), reaching values similar to those observed in control cells either for inward currents (*p* = 0.20, *n* = 4), or outward currents (*p* = 0.15, *n* = 4). However, neither stimulation by IBMX during time course of the patch-clamp experiments, nor addition of cAMP into the patch pipette changed membrane conductance of the cells overexpressing PfPKA-R cells (data not shown).

**Figure 6:**
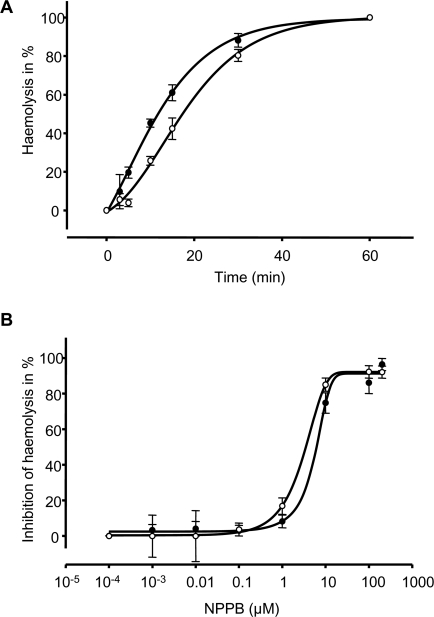
Haemolysis of *pfpka-r* Transformed Parasites (A) Time course haemolysis of *pfpka-r* parasites (○, *n* = 3) and control parasites (•, *n* = 3) suspended in iso-osmotic solution of sorbitol. (B) Dose-response curves for NPPB on the sorbitol haemolysis of *pfpka-r* transformed parasites (○, *n* = 3) and control parasites (•, *n* = 3).

In order to test whether PfPKA-R overexpression could interfere with NPP activity, semi-quantitative haemolysis experiments were carried out using sorbitol as permeating substrate through NPPs. In parasites overexpressing PfPKA-R permeability to sorbitol appeared reduced, since t_1/2_ of lysis was significantly increased (18.2 ± 1.6 min, *n* = 3) compared to what is observed in control cells (12.7 ± 0.8 min, *n* = 3, *p* = 0.03, Figure 6A). However, sensitivity to the anion carrier inhibitor NPPB was not altered, since IC_50_ measurements in our conditions are similar in parasites overexpressing PfPKA-R and in control parasites (4.5 ± 1 μM and 7 ± 1 μM respectively *n* = 3, Figure 6B).

## Discussion

In contrast to mammalian cells that have several PKA-C and PKA-R subunits, we show here that cAMP signalling in P. falciparum is extremely reduced, as the parasite possesses only a single regulatory subunit in addition of its single PKA-C subunit. Overexpression of the regulatory subunit in transgenic parasites, or addition of recombinant PfPKA-R or PKI to the pipette in patch-clamp experiments, significantly down-regulates the conductance of one of the erythrocyte plasma membrane anion channels that we have previously described at the single channel level [36]. In this context, it is worth noting that no gene encoding PKI can be detected in the P. falciparum genome, although we have shown previously that P. falciparum PKA-C activity is somewhat PKI sensitive [22]. One of the P. falciparum adenylate cyclases that have been reported has a putative N-terminal ion channel domain encoding a canonical S4 voltage sensor [37]. This leads to the intriguing possibility that cAMP signalling in malaria parasites may regulate anion conductance at the plasma membrane. Membrane currents measured in this study arise from the activity of two different types of chloride channel [13]: besides the activity of the PKA-dependent chloride channel, another type of chloride channel belonging to the ClC-2 family is constitutively active in infected cells. Although the possible activation of this channel type by phosphorylation is still controversial [38,39], this could explain why addition of PfPKA-R failed to completely inhibit anion transport.

Transgenic parasites that overexpress the regulatory subunit have a reduced growth (Figure 5A) and normal growth rate is largely restored upon enhancement of intracellular cAMP levels (Figure 5B and 5C). This is consistent with our previous observation that H89, which inhibits PfPKA kinase activity *in vitro,* blocked parasite growth [22] and the more recent report that the parasite cell cycle is also regulated by cAMP [25]. This could also explain why changes in intracellular cAMP concentrations following addition of IBMX, seems to be lethal for the parasite. During erythrocyte development cAMP levels appear to be finely regulated, as over-stimulation of cAMP production tends to the accumulation of parasites at the schizont stage [25]. Furthermore, IBMX, is poorly selective between cAMP phosphodiesterases and cGMP phosphodiesterases, so we cannot exclude the possibility that the effects observed in the presence of IBMX are due to increases in intracellular concentrations of other cNMPs, as has been shown for exflagellation [40,41], or the growth defect observed in the presence of 8-Bromo-cGMP (ED_50_ 10 μM) [30]. P. falciparum codes for four different phosphodiesterases, but only one (PfPDE1) has been characterised and, shows no cAMP hydrolytic activity up to 5 mM cAMP, but has 135 time higher cGMP hydrolysing activity without any competition between cAMP and cGMP [30]. In addition, nor can we rule out the possibility that the effects of cAMP agonists (IBMX and 8-Br-cAMP) observed in the present study are a consequence of a rise in intracellular Ca^2+^ concentrations, as described by Beraldo *et al.* [42]. Indeed, it is known that modulation of Ca^2+^ signalling can be modulated by a cAMP-dependent pathway and some evidence suggests an important role for Ca^2+^ in the control of key processes in *Plasmodium* such as genes expression and cell cycle progression [25,43]. Therefore, it is possible that PfPKA regulates parasite growth via other factors independent of anion channel conductance, as the lack of a similar channel in erythrocytes from cystic fibrosis patients does not seem to interfere with parasite growth [14,16]. Moreover, repeated attempts to disrupt the PKA regulatory or catalytic domain in P. berghei, the rodent parasite, failed, suggesting the crucial role of the protein for the malaria parasite (Tewari, Moon and Billker, pers. com.).

In patch-clamp experiments on erythrocytes infected by PfPKA-R overexpressing parasites, the addition of non-permeable cAMP in the patch-clamp pipette does not release the inhibition of anion conductance, whereas 24 h IBMX stimulation restore the membrane conductance to the level of controlled cells. This raises the question of the localisation of the PfPKA-R. Previous studies have shown that a PEXEL/HT motif promotes export of proteins from the parasite into the host cell [44,45]. PfPKA-R and PfPKA-C sequences do not have a recognisable PEXEL/HT motif, but a parasite antigen (PfEMP1) present in the red blood cell plasma membrane and the kinase PfGSK-3 [46], which is addressed to the Maurer's cleft also lack recognisable export motifs [47].

cAMP in infected cells is most likely synthesized in the parasite cytosol and cAMP is normally membrane impermeable. However, we cannot rule out that some parasite-derived cAMP might get into the erythrocyte cytosol via the non specific channel that can pass soluble macromolecules of up to 1400 Da and functions as a high capacity, low affinity molecular sieve [48]. We entertain here, the possibility that overexpression of the PfPKA regulatory subunit leads it to act as a sink for cAMP thereby reducing available cAMP levels, both within the parasite and eventually in the erythrocyte cytosol. In this scenario, addition of recombinant MBP-PfPKA-R would limit the release of monomeric active PKA catalytic subunit (host PKA-C, or secreted PfPKA-C), and thus the activation of subsequent cellular mechanisms such as the activation of the anion transport.

The delay observed in haemolysis for the parasites overexpressing PfPKA-R, together with and the complete inhibition of membrane currents by unspecific dephosphorylations suggest that some NPP activity is under the dependence of phosphorylation via cAMP-dependent kinases, either directly or indirectly. In the absence of compelling localization data for PfPKA-R and PfPKA-C, we cannot exclude that the effect of PfPKA-R on anion conductance at the erythrocyte membrane may be mediated indirectly, through PKA-dependent regulation of other effectors. In this respect, it is worth mentioning that several protein kinases and phosphatases have been shown to be targeted to the erythrocyte and cAMP-dependent PKA may be involved in their activation and/or secretion [46,49,50].

It has been known for some time that non-infected erythrocytes have a PKA activity and regulatory RI and RII subunits, with the RI subunit being associated with the plasma membrane [51]. PKA activity at the plasma membrane could contribute to the low-level anion channel conductance of non-parasitized red blood cells [8]. In extracts from P. falciparum-infected erythrocytes host cell PKA-C appears to be cleaved, although the physiological significance of this observation has not been established [22]. Although a role for PfPKA-C in the regulation of anion channels seems likely, we cannot exclude the possibility that host PKA-C contributes to induction of anion conductance in P. falciparum-infected red blood cells. In this scenario, erythrocyte PKA-C would become active at the schizont stage, perhaps following a parasite-dependent increase in cAMP levels in the red blood cell cytosol. At the red blood cell plasma membrane erythrocyte PKA-C is associated with the RI subunit and the recent report that RI can bind integrin α4β1 and recruit PKA-C to phosphorylate the integrin's cytoplasmic tail [52] opens up a wealth of new possibilities for parasite-modulation of integrin function in malaria. Even though erythrocytes do not express α4β1 one possibility that we are currently testing is that the R subunit (host and/or parasite) binds directly to another integrin and regulates anion channel conductance in an integrin-dependent fashion, as a role for β1integrin in anion channel conductance has been reported for ventricular myocytes [53]. Clearly, other integrin-dependent signalling pathways in P. falciparum-infected hepatocytes and erythrocytes could be also modulated by PKA, whether it be of erythrocyte or parasite origin.

## Materials and Methods

### Cloning and expression of PfPKA-R.

Primers were designed for amplification and cloning of the entire ORF of 1326 bp (PlasmoDB identifier PFL1110c) into the pMALc2X vector (New England Biolabs). The primers contained *Eco*RI (forward primer: CCGGAATTCATGGGCAATGTGTGCACATGG), or *Hin*dIII (reverse primer: CCCAAGCTTTTAATTTTCATCAATACAA-GTTG) restriction sites (single underline). After amplification from a P. falciparum cDNA library (kindly provided by Alister Craig) with a *Taq* DNA polymerase (Takara), the PCR product was digested with *Eco*RI and *Hin*dIII prior to insertion in the pMALc2X vector (New England Biolabs). The pMALc2X*–pfpka-r* plasmid was electroporated into E. coli BL21, and the insert was verified by sequencing prior to expression of the recombinant protein. Protein expression was induced for 3h at 37°C with 0.3 mM isopropyl-α-D-galactoside (IPTG), after the 250 ml culture has reached an OD_600_ value of 0.6 in 2xYT medium with 100 μg/ml ampicillin. All purification steps were performed at 4°C. Bacterial pellets were lysed with lysosyme and by sonication in 5 ml of lysis buffer (50 mM Tris pH7.5, 50 mM NaCl, 5 mM EDTA, 1 mM phenylmethylsulfonyl fluoride [PMSF] and Complex^TM^ protease inhibitors protease inhibitor tablet from Roche Molecular Biochemicals). Lysates were cleared by centrifugation (11,000 rpm, 4°C, 30 min) and the soluble fraction was incubated for 90 minutes at 4°C under mild agitation with 0.5 ml of amylose beads (New England Biolabs). The slurry was washed in lysis buffer and the MBP-PfPKA-R fusion protein was eluted with elution buffer (50 mM Tris pH8.0, 50 mM NaCl, 0.1 mM EDTA, 1 mM PMSF, Complex^TM^ protease inhibitors protease inhibitor tablet and 12 mM maltose).

### Parasite extracts.

P. falciparum 3D7 maintained in a modified erythrocyte culture were synchronized by 5% sorbitol. The parasites were released from infected erythrocytes by saponin (0.1% w/v) lysis and P. falciparum pellets were sonicated in RIPA buffer (30 mM Tris, pH8.0, 150 mM NaCl, 20 mM MgCl2, 1 mM EDTA, 1 mM dithiothreitol, 10 μM ATP, 0.5% Triton X-100, 1% Nonidet P-40, 10 mM β-glycerophosphate, 10 mM NaF, 0.1 mM sodium orthovanadate, 1 mM phenylmethylsulfonyl fluoride, 10 mM benzamidine, and Complex^TM^ protease inhibitors). The lysates were cleared by centrifugation (15,000 rpm for 15 min at 4°C), and the total amount of proteins in the supernatant was measured by a Bio-Rad protein assay, based on the method of Bradford, using BSA as a standard.

### Kinase assays.

PKA activity was assayed by measuring ^32^P incorporation into a synthetic peptide (Kemptide L-R-R-A-**S**-L-G, Promega), as described previously [22]. Briefly, in a standard volume (30 μl) containing (50 mM MOPS pH7, 0.5 mM MgCl_2_, 40 μg/ml BSA, 50 mM NaF, 1 mM β glycerophosphate, 1 mM PMSF and Complex TM mixture tablets Roche Molecular Biochemicals), reactions were initiated by addition of 300 μM Kemptide and 15 μM ATP / 5 μCi of (γ-^32^P)-ATP. The reactions were performed in presence or absence of 40 μM cAMP (prior incubation at 30°C for 15 min), and in presence or absence of recombinant MBP-PfPKA-R (or MBP as a control). Significance was assessed using the Fisher *F* test and Student's t-test.

### Northern blot analysis.

Approximately 5 micrograms of total RNA were electrophoresed in a 1% agarose/formaldehyde gel. After RNA visualisation with ethidium bromide staining, which confirmed that equivalent amounts of RNA were loaded in the two samples, gel was blotted to Hybond-C nitrocellulose (Amersham). Filters were cut, and hybridised with ^32^P-labelled probes specific for the *pfpka-c* and the *pfpka-r* genes, respectively. 735 bp PCR fragment for *pfpka-c*, and the whole *pfpka-r* gene (1326 bp) were amplified with the following primers: CATGGATCATTCAAAGATGAC (PfPKA F2); GGAAGATCTCTACCAATCATAAAATGGATCATTTTC (PfPKA *Bgl*II stop R); CCGGAATTCATGGGCAATGTGTGCACATGG (PfPKA SUR *Eco*RI F); CCCAAGCTTTTAATTTTCATCAATACAAGTTG (PfPKA SUR *Hin*dIII R). Hybridisation signals were revealed after autoradiographic exposure (40 h).

### Construct for P. falciparum transfection.

Oligonucleotides were designed for amplification containing *Pst*I and *Hin*dIII restriction sites (single underline in primers) (forward primer: ATACTGCAGATTATGGGCAATGTGTGCACATGG; reverse primer: ATAAAGCTTAACGACGGCCAGTGCCAAGCT). After amplification, PCR products were restriction digested with *Pst*I and *Hin*dIII prior to insertion in the expression vector pHL*-dhfr* [50].

### Parasite transfection and PfPKA-R overexpression.

The entire *pfpka-r* coding region was amplified from cDNA and cloned into the transfection plasmid pHL*-dhfr*, a derivative of pHLH1 vector [54]. pHL*-dhfr-luciferase* includes a luciferase open reading frame (*luc*) flanked by the *P. falciparum 5′hrp3* promoter and the *3′hrp2* terminator. The vector contains the human dihydrofolate reductase sequence (*dhfr*), which allows selection of stable transformed parasites NF54 isolate (from which the 3D7 line is derived) by addition of 40 ng/ml pyrimethamine in the culture medium. The pHL*-dhfr-pfpka-r* plasmid differs from the pHL*-dhfr-luciferase* plasmid by replacement of the luciferase open reading frame by that of *pfpka-r*. Parasites were transfected, as previously described [55]. Briefly, uninfected human erythrocytes were electroporated with 50 μg of plasmid DNA in complete cytomix medium (120 mM KCl ; 0.115 mM CaCl_2_ ; 5 mM MgCl_2_ ; 5 mM K_2_HPO_4_ ; 5 mM KH_2_PO_4_ ; 2 mM EGTA and 30 mM Hepes pH7.6 adjusted with NaOH) using a Bio-Rad gene pulser and 0.2 cm cuvettes (conditions 0.31 kV and 960μF). Erythrocytes from two electroporations were combined for each 5 ml culture and inoculated with late stage parasites purified using a Percoll/sorbitol. Validation of the transfection was done by Q-PCR. After transfection, and the selection by pyrimethamine, more than two months were necessary to recover significant amount of parasites. Moreover, due to the defect of growth of the *pfpka-r* transformed parasites, the cultures were maintained at the beginning with a 2.5% hematocrit instead of the 5% hematocrit generally used, in order to ensure the possibility of adding fresh blood more frequently, improving the fitness of the culture and the relative amount of infected cells.

### RNA preparation and real-time PCR assays of gene transcription.

Prior RNA extraction, synchronized infected RBCs (Rings, trophozoites and schizonts) were washed 2 times in 1× PBS, permeabilized with 0.05% saponin, and then pelleted parasites were rapidly washed 3 times with 1X PBS. RNA extraction was performed with the TRIZOL LS Reagent® as previously described [56]. RNA to be used for cDNA synthesis was treated with Deoxyribonuclease I® (Invitrogen) as described by the manufacturer. A total of 1600 ng of RNA was digested in a 20 μl reaction. Samples were incubated at room temperature for 30 minutes followed by 10 minutes heat inactivation at 65°C. cDNA synthesis was performed with Superscript II Rnase H reverse transcriptase ® (Invitrogen) with random primers ® (Invitrogen) as described by the manufacturer. cDNA was synthesized from 800 ng RNA in a 40 μl reaction. For each cDNA synthesis reaction, a control reaction without reverse transcriptase was performed with identical amounts of template.

Gene specific primers were designed for the *pfpka-c* (PFI1685w ) and *pfpka-r* genes using primer 3 software (http://frodo.wi.mit.edu/cgi-bin/primer3/primer3_www.cgi) (forward primer :TTAATGACGACGGTTCAAGC [PfPKAr_qf]; reverse primer : TCCAGTCACCATATGCTTCG [PfPKAr_qr] and forward primer : TGGATGTTTTTATGCAGCTCAG [PfPKAc_qf]; reverse primer TCCATGTCCGACGTTCAATA[Pf©PKAc_qr]). With this primer set 153 bp fragments of *pfpka-r* gene and 223 bp fragments of *pfpka-c* present in the NF54 Plasmodium falciparum strain are generated. Amplification efficiency was verified by testing all primer pairs using a two 3 log range of concentrations of genomic DNA obtained from transformed parasites and two control genes, namely *arginyl-tRNA synthetase* (PFL0900c) and *seryl-tRNA synthetase* (PF07_0073) kindly provided by T.J. Templeton and C. Lavazec. Real-time PCR was performed using an ABI Prism 7900HT sequence detector (Applied Biosystems). Reactions were prepared in 25 μl volume using SYBR Green PCR master mix (Applied Biosystems) and 1 μM primers. Specificity of amplification was confirmed by melting-curve analysis for each PCR product. Triplicate PCR were analyzed for each sample. The genes transcriptions were expressed relative to the expression of a single copy housekeeping gene (ΔCt method). Briefly, the ΔCt for each individual primer pair was determined by subtracting the measured Ct value from the Ct value of the control *seryl-tRNA synthetase* (User bulletin 2, Applied Biosystems, http://www.appliedbiosystems.com/). ΔCts were then converted to relative copy numbers with the formula 2^ΔCt^.

### Plasmodium cell cycle.

The effects of drugs on the growth of *in vitro* parasite cultures were determined as follows. Erythrocyte suspensions with ring-stage synchronized parasites were distributed in triplicate into 24-well plates at 2% parasitemia and 2% hematocrit. The incubations were performed for 72 h at 37°C in a closed chamber with controlled atmosphere in stationary condition. The proportion of different parasite stages as well as parasitemia was determined by analyzing 1,000 RBCs on Giemsa-stained slides. As a negative control, cultures treated with solvent only (DMSO) were assayed. The total parasitemia was an arithmetic sum of rings, trophozoites, and schizonts at each time point.

### Patch clamp experiments.

The whole-cell configuration of the patch-clamp technique was assessed by the development of small capacitance transient and reduction of access resistance. Cation movements across the membrane from the exterior (bath) to cytoplasmic side is defined as inward current and shown as downward deflection in whole cell recordings. Seal resistances were 4–20 GΩ.

Patch pipettes (tip resistance 10–20 MΩ) were prepared from borosilicate glass capillaries (GC150 TF-10, Clark Medical Instruments, PHYMEP, France) pulled and polished on a Werner Zeitz DMZ programmable puller (Augsburg, Germany). The ruptured patch whole-cell configuration was used to record whole-cell currents. Whole-cell currents were recorded using a RK400 (Biologic, France) amplifier, with voltage command protocols generated and the currents analyzed using the WCP Software (WCP V3.3.3. Software, Strathclyde, UK) by evoking a series of test potentials (*V*T) from −100 to +100 mV in 10 mV steps for 500 ms from a holding potential (*V*H) of 0 mV. Data for the construction of *I–V* curves were the mean current measured between 200 and 400 ms. Data are given as mean values ± S.E.M. Significance was assessed using the Fisher *F* test and Student's t-test. In all cases, *n* denotes the number of cells tested.

Different bath (150 mM NMDG-Cl, 1.4 mM CaCl_2_, 1 mM MgCl_2_, 15 mM Hepes, 10 mM glucose, pH7.4, 320 ± 5 mOsmol, pCa3) and pipette (150 mM NMDG-Cl, 0.29 mM CaCl_2_, 1.22 mM MgCl_2_, 5 mM EGTA, 10 mM Hepes-Tris, 10 mM glucose, pH7.2, 320 ± 5 mOsmol, pCa7) solutions were used to ensure proper free calcium concentrations at both sides of the membrane. Since temperature has no influence on whole cell currents in infected red blood cells all experiments were performed at room temperature.

### Reagents used in patch-clamp experiments.

RBCs were obtained from healthy volunteers, who gave informed, written consent, in accordance with the Declaration of Helsinki, cAMP (cyclic adenosine-monophosphate), alkaline phosphatase, 8Br-cAMP (8-Bromide-cyclic adenosine-monophosphate), PKI (Protein Kinase Inhibitor), NMDG Cl (N-methyl-D-glucamine chloride), IBMX (isobutylmethylxanthine) and NPPB (5-nitro-2-(3-phenylpropylamino)benzoic acid) were purchased from Sigma (Saint Quentin Fallavier, France).

### cAMP enzyme immunoassay.

P. falciparum-infected cells were submitted to 24 h of IBMX treatment. Following treatment cells were washed twice in 1X PBS and analysed with the cAMP immunoassay kit (GE Healthcare, Amersham Biotrak) following protocol N°3.

### Haemolysis experiments.

For standard semi-quantitative haemolysis assays, haemoglobin release was used to estimate lysis time, as previously described [57]. Culture suspensions (2–5% parasitaemia) were washed three times in culture medium without serum and resuspended at 50% hematocrit.

Time courses started with the addition of a 0.4 ml aliquot of cell suspension to 3.6 ml of the sorbitol iso-osmotic solutions (300 mM sorbitol, 10 mM hepes, 5 mM glucose, pH 7.4) to give a cell concentration of approximately 0.5.10^8^ cells/ml. Experiments were performed in triplicates. At predetermined intervals (0, 3, 5, 10, 15, 30, 60 min) 0.5 ml aliquots of the suspension were transferred to microcentrifuge tubes containing 0.5 ml of an ice-cold “stopping solution” (400 mM sucrose in H_2_O). The tubes were centrifuged for 30 seconds then 0.9 ml of the supernatant solution was transferred to another tube for the subsequent spectrophotometric estimation of haemoglobin concentration by absorption at a wavelength of 540 nm (A_540_). In all such experiments the A_540_ value corresponding to full haemolysis of trophozoite-infected erythrocytes was estimated from the final A_540_ value achieved in the supernatant solution from infected cells suspended in an iso-osmotic sorbitol.

The percent lysis values at different times were fitted by nonlinear regression using equation for a sigmoidal shape according the equation *y* = *a*/(1+exp(−(*x*−*x*_0_)/*b*)), where *y* is the percent lysis value, *a* is the maximal lysis, *x* is the sampling time, *x*_0_ is essentially the *t*_1/2_ of lysis, and *b* represents the variability of cells in the population [58]. The *t*_1/2_ of lysis calculated for each experiment was then compared using a paired two-tailed Student's t-test.

When drugs were tested, the percentage of inhibition was determined relative to non-treated cells when haemolysis was at maximum.

## Supporting Information

Figure S1 Phylogenetic Tree of cAMP-Dependent Kinase Regulatory Subunits Constructed Using the “Neighbours Joining” (N-J Tree) Algorithm Included in CLUSTALX The tree includes the Type I alpha, Type I beta, Type II alpha, and Type II beta human PKA regulatory subunits, as well as the regulatory subunits of Euplotes octocarinatus, Drosophila melanogaster, and P. falciparum.
(2.8 MB TIF)

Figure S2 IBMX Stimulation of Membrane Anion Conductance of *pfpka-r* Transformed Parasites (A) Slope conductance for the outward current calculated by linear regression between +20 and +100 mV on current-voltage relationships for control cells, *pfpka-r* transformed parasites, and *pfpka-r* transformed parasites stimulated 24 h with IBMX (500 μM).
(B) Slope conductance for the inward current calculated by linear regression between −100 and −40 mV on current-voltage relationships for control cells, *pfpka-r* transformed parasites, and *pfpka-r* transformed parasites stimulated 24 h with IBMX (500 μM).
Data are means ± standard error of the mean; *n* = 3–5. *, significant difference between membrane conductance calculated for control cells and *pfpka-r* transformed parasites or significant difference between membrane conductance calculated for *pfpka-r* transformed parasites and *pfpka-r* transformed parasites stimulated with 500 μM IBMX (*p* < 0.05, two-tailed t-test); ns, no significant difference between control cells and *pfpka-r* transformed parasites stimulated with 500 μM IBMX (two-tailed t-test).
(4.1 MB TIF)
